# Relating consequential falls to individual and combinations of prescription medication use in Swedish older adults

**DOI:** 10.1007/s00228-026-04000-2

**Published:** 2026-02-06

**Authors:** Daniel S. Peterson, Linda Johansson, Bjorn Westerlind, Thais Lopes de Oliveira, Deborah Finkel

**Affiliations:** 1 Institute of Gerontology, School of Health and Welfare, Jönköping University, Jönköping, Sweden; 2 College of Health Solutions, Arizona State University, Phoenix, AZ USA; 3 Department of Health, Medicine, and Caring Sciences, Linköping University, Jönköping, Sweden; 4 Department of Geriatrics, County Hospital Ryhov, Region Jönköping County, Jonkoping, Sweden; 5 Department of Medical Epidemiology and Biostatistics, Karolinska Institutet, Stockholm, Sweden; 6 Center for Economic and Social Research, University of Southern California, Los Angeles, CA USA

## Abstract

**Purpose:**

We assessed the relationship between prescription of medications (both individually and in combination) and consequential falls.

**Methods:**

Medication prescription and prospectively registered falls over 2.5 years were extracted from 441 participants within a Swedish National Quality Register. Conditional generalized estimating equations, considering dependence of longitudinal data as a cluster to correct confidence intervals, were used to relate medications (individual and in combination) and falls.

**Results:**

Regarding individual-medications, Angiotensin II receptor blockers and diuretics (C09DA) were significantly related to incidence of consequential falls (*p* = 0.022). Six drug combinations significantly related to fall incidence. Most frequently, falls were observed when platelet aggregation inhibitors (B01AC) were prescribed with C09DA, opioids (N02AA), or blood glucose lowering drugs (biguanides, A10BA).

**Discussion:**

Caution should be taken when prescribing cardiovascular medications. Further, prescription of platelet aggregation inhibitors could increase the incidence of a negative outcome of a fall when prescribed in people at risk for falls.

**Supplementary Information:**

The online version contains supplementary material available at 10.1007/s00228-026-04000-2.

## Key points

The relationships between combinations of medications and rates of injurious falls is poorly understood.

We addressed this gap using prospectively collected falls and medication data from a Swedish National Registry.

Cardiovascular and platelet aggregation inhibitor medications related to consequential falls in this well-characterized cohort of older adults.

**Supplementary Information:**

The online version contains supplementary material available at 10.1007/s00228-026-04000-2.

## Introduction

Falls are the most frequent cause of injury and injury death among people over age 65 (Kakara et al. 2023), and with over 684 000 deaths every year falls are considered a public health issue (World Health Organization 2024). Falls can be variably impactful to a person’s life. In fact, most falls do not cause injury. Rather, approximately 20–30% of falls among adults 65 years or older result in moderate to severe injuries (Hamedan Al Maqbali 2014). However, these injurious falls can have a particularly negative impact on quality of life. Specifically, injurious falls have numerous societal and personal consequences, including an abrupt reduction in physical functioning, bodily pain, and social functioning (Hartholt et al. 2011; Peeters et al. 2015). As such, while all falls should be considered potentially detrimental to quality of life, special attention should be paid to the predictors of injurious falls, or those that result in a physical consequence.

While causes of falls are multifactorial, medications can have a pronounced effect on fall-risk. Considerable work has been conducted to identify potentially inappropriate medications (PIMs) for older adults. In particular the Beers criteria by the American Geriatric Society has outlined such medications (Panel., 2023). Among these PIMs are medications that specifically increase fall risk (Fall Risk Increasing Drugs, FRIDs), including diuretics, antipsychotics, antiepileptics, cardiovascular drugs, analgesics, among others (Seppala et al. 2021; van der Velde et al., 2023). In addition to these fall-risk increasing drugs, use of certain medications may be related to falls, albeit in a non-causal way. For example, increased folic acid use was shown to relate to increased fall-risk (Shahar et al. 2009), perhaps because it was prescribed in those with reduced nutrition- the direct cause of the fall-risk. Further, commonly used cardiovascular prevention strategies in older adults such as low-dose aspirin have been questioned due to risk of major bleeding (McNeil et al. 2018) and is also shown to be associated with an increased risk of severe injuries when falls occur (Barker et al. 2022).

A notable gap in the current literature is understanding how drugs may interact to increase the risk of falls and consequences of falls. Considerable work has shown that polypharmacy, in general, does increase fall risk (Dhalwani et al. 2017; Reinhild Haerig et al. 2023; Zaninotto et al. 2020; Zia et al. 2015), however it is unclear whether specific combinations of drugs increase this risk or, rather, if increased fall risk is due to the increased comorbidities typically associated with polypharmacy. As such, additional work is necessary to assess how combinations of specific medications may relate to falls. Further, some previous work exhibits methodological limitations including (1) cross-sectional or retrospective design (often utilizing recall to identify falls), (2) small samples, (3) single-center designs, and (4) relatively short follow-up periods (Hien le et al. 2005; Ryan-Atwood et al. 2017; Wang et al. 2021). In particular, longitudinal, prospective analyses that link medication prescription to fall incidence are important to increase the likelihood of a meaningful link between these factors.

The current study aimed to extend knowledge regarding the medication-falls relationship by associating single medications and combinations of medications to incidence of consequential falls in older adults. We extend previous work in several ways, including (1) utilization of a large and well-characterized longitudinal database of Swedish residents with robust prescription data information and prospectively reported falls, (2) inclusion of all medications (that met minimum prescription frequency requirements) rather than only previously identified FRIDs, and (3) utilized a within-subject design to assess how each medication (individually and in combination with another medication) did or did not relate to fall incidence.

## Methods

### Participant selection

National health quality registers are collected in Sweden, providing population-based information on health outcomes. To assess the relationship between medication use and falls, we consolidated data from 3 separate registries: (1) the Prescribed Drug Registry (to identify medication use), (2) the national preventive care quality register for older adults called Senior Alert (Edvinsson et al. 2015) (see also: https://www.senioralert.se/) which, from 2015 to 2017, tracked falls (Trinks et al. 2018), and (3) the Cause of Death Registry (to identify people who passed away over the 2015–2017 window). A Swedish twin population (Screening Across the Lifespan of Twins; SALT(Lichtenstein et al. 2006) cohort (*n* = 44816) was used to link data across these registers. Data was linked in 2014, and a second linkage was performed in 2017. In the present study, only data from the second linkage is used, i.e., data registered between 2015 and 2017. Ethical approval for the research project was provided by the Swedish EthicalReview Authority in Linköping, Sweden (dnr: 2014-2635-271; 2017-549-32; 2020-04345; prior to 2020 known as the Regional Ethical Review Board; Linköping) andfollowed the guidelines outlined in the Helsinki declaration (World MedicalAssociation, 2013). Informed consent was not obtained as the study was based onpseudonymized data from a national quality registry. According to Swedish Adults regulations, individual consent is not required for registry-based research. However,the participants have given their informed consent for their information to becollected by the Senior Alert register and used for quality improvement, research,and healthcare development. No funding was used to support this work. Clinical trialnumber: not applicable.

From 2015 to 2017 (when falls data were captured), the Senior Alert registry followed a total of 2903 individuals with relatively high levels of frailty (Johansson et al. 2021). Falls were prospectively registered by health care professionals in one of the following types of care setting: hospital ward, primary care centers, home health care or nursing homes (Trinks et al. 2018). In addition to noting that a fall occurred, care staff also noted whether a ‘consequence’ occurred as a result of the fall. Consequences included any of the following: death, concussion, head injury, vertebral damage, prolonged hospital or external care, soft tissue injury, wounding, other injury, or fracture of any of the following: arm, foot, leg, hip or thigh. In the present analysis, a fall with any in which any of these circumstances was recorded was flagged as a “consequential fall”.

Of the 2903 individuals in the Senior Alert registry, 440 people had at least 1 registered fall and complete medication data to be included in the current analysis. This population was utilized for the current analysis, as they represented a group with confirmed falls tracking. Next, we searched the Prescribed Drug Registry to identify all medication prescription (date and type of medication) across time in the 440 people with registered falls. Medications were listed via the World Health Organization (WHO) Anatomical Therapeutic Chemical (ATC) classification system (https://atcddd.fhi.no/atc_ddd_index/). Five-digit ATC codes of each medication were used to identify prescriptions.

Co-morbidities were not reliably included in the available registries. However, the total number of medications was available. This outcome tracks closely with overall health and likelihood of falls (Maher et al. 2014) and, as noted below, was included in statistical models to account for possible differences in overall health over time that could obscure relationships between individual (or combination) medications and falls.

### Analytic approach

A within subject approach was utilized to identify relationships between the prescription of medications and registered falls. While there are pros and cons to such an approach (Jung et al. 2023), it was chosen for the current analysis to increase statistical power of our models and utilize the high temporal resolution of medication prescription and falls in the current dataset. Further, this approach facilitates accounting for possible unmeasured, time-stable confounding variables.

To build this model, we first separated the observation period for each participant (mean 2.4 years, see Table 1) into 30 day windows. This window was chosen to be long enough to identify both a fall and a prescription in each window, while being short enough to provide a more direct link between medication use and falls. Utilization of longer windows, up to 1 year in some previous studies (Ming et al. 2022) could reduce the temporal relevance of the medication-fall relationship, particularly for medications that are used acutely. Then, for each window, we noted whether (1) a consequential fall occurred, and (2) whether each medication (or 2-medications together for combination analyses, see details below) were prescribed. Conditional Generalized Estimating Equation (cGEE logistic self-comparison models), were then used to assess if there was concordance (i.e., incidence of fall and medication prescription) across each time-window between an instance of a registered fall and prescription of a medication (Goetgeluk and Vansteelandt 2008; Lopes De Oliveira et al., 2024; Tang et al. 2023). The cGEE models considered the dependence of the individual’s longitudinal data as a cluster to correct confidence intervals and only considered variation in medication intake for fall prediction. Separate models were run for each medication (or combination of medications, see below) included in the analysis, and the Benjamini-Hochberg (BH) false discover rate (FDR) correction was applied to individual-medication and combination-medication analyses to reduce the likelihood of type I error.

**Table 1:** Fall and participant characteristics

	Male (*N* = 167)	Female (*N* = 273)	Total (*N* = 440)	*p* value
All falls				0.142
Sum	762	1036	1798	
Mean (SD)	4.6 (5.3)	3.8 (5.3)	4.1 (5.3)	
Median (Q1, Q3)	2.0 (1.0, 5.0)	2.0 (1.0, 4.0)	2.0 (1.0, 4.0)	
Range	1.0–32.0	1.0–54.0	1.0–54.0	
Consequential Falls				0.887
Sum	181	290	471	
Mean (SD)	1.1 (1.5)	1.1 (1.6)	1.1 (1.5)	
Median (Q1, Q3)	1.0 (0.0, 1.0)	1.0 (0.0, 1.0)	1.0 (0.0, 1.0)	
Range	0.0–9.0	0.0–12.0	0.0–12.0	
Number of Medications Prescribed				0.828
Mean (SD)	16.8 (7.1)	16.7 (7.0)	16.7 (7.0)	
Median (Q1, Q3)	16.0 (12.0, 20.5)	16.0 (12.0, 21.0)	16.0 (12.0, 21.0)	
Range	1.0–52.0	3.0–43.0	1.0–52.0	
Age				< 0.001
Mean (SD)	81.1 (8.2)	85.4 (7.1)	83.8 (7.8)	
Median (Q1, Q3)	82.0 (75.8, 87.0)	86.0 (81.0, 90.9)	85.0 (79.0, 89.0)	
Range	59.0–98.0	61.9–99.0	59.0–99.0	
Recurrent Faller Status				0.298
No	56 (33.5%)	105 (38.5%)	161 (36.6%)	
Yes	111 (66.5%)	168 (61.5%)	279 (63.4%)	
Observation Period				0.032
Mean (SD)	2.3 (0.9)	2.5 (0.7)	2.4 (0.8)	
Median (Q1, Q3)	2.9 (1.8, 2.9)	2.9 (2.2, 3.0)	2.9 (2.1, 3.0)	
Range	0.0–3.0	0.1–3.0	0.0–3.0	

To ensure that cGEE models had a sufficient number of observations to converge, analyses were only run for medications with at least 250 prescriptions across all participants and temporal windows. There were 288 unique medications prescribed across all participants and time-windows. However, only 55 were prescribed at least 250 times (across participants and windows), and thus were included in the individual-medication analysis. For combination analyses, we identified instances where any 2 of these medications were both prescribed within a 4-week window. This created 1496 medication combinations, however of these, only 163 medication combinations were prescribed > 250 times. As with the individual medication analysis, within-subject cGEE models were then run on these 163 medication combinations (see Supplemental Fig. 1 for flow-diagram of medications included in each analysis).

For both individual medication and combination medication analyses, cGEE models were controlled for age and the total number of medications prescribed over that time window to partially control for current health condition.

## Results

### Participant and fall descriptives

**Fig. 1:**
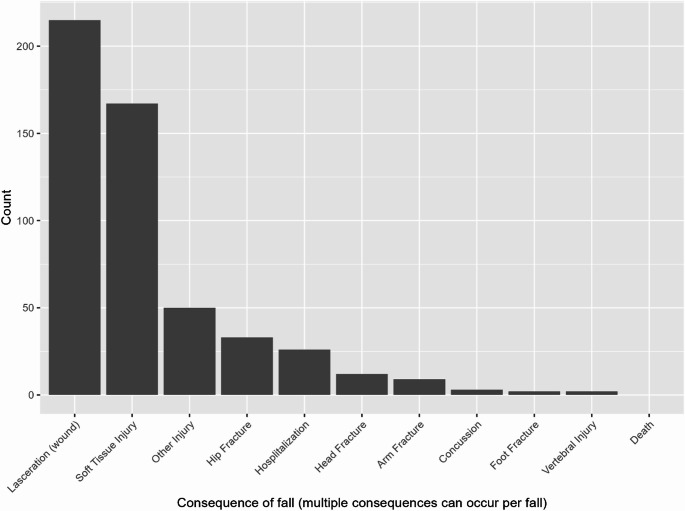
Histogram of the rates of observed consequences associated with falls

Participant and fall descriptives are shown in Table 1, and a histogram of the number of individuals experiencing consequential falls is provided in Supplemental Fig. 2. The 440 participants included in the study were 83.8 years at the time of enrollment, on average, and were tracked for an average of 2.4 years. Within the study period, participants experienced 1798 falls, of which 471 were noted to have any reported “consequence”. The median number of consequential falls was 1. The distribution of consequences reported for these falls is shown in Fig. 1; the two most common types of fall consequences were laceration and soft tissue injury.

**Table 2:** Medication combinations observed to be statistically significantly related to fall incidence after (1) controlling for age & total number of medications, and (2) FDR correction for multiple comparisons. ATC-code/drug descriptions are provided in the text

Medication 1	Medication 2	FDR-corrected *p*-value
B01AC	N02AA	0.020
	C09DA	0.002
	A10BA	0.001
C09DA	B03BB	0.001
C03CA	H02AB	0.013
A12AX	H03AA	1.53E-06

### Relationship between consequential falls and medications (individually and in combination)

**Fig. 2:**
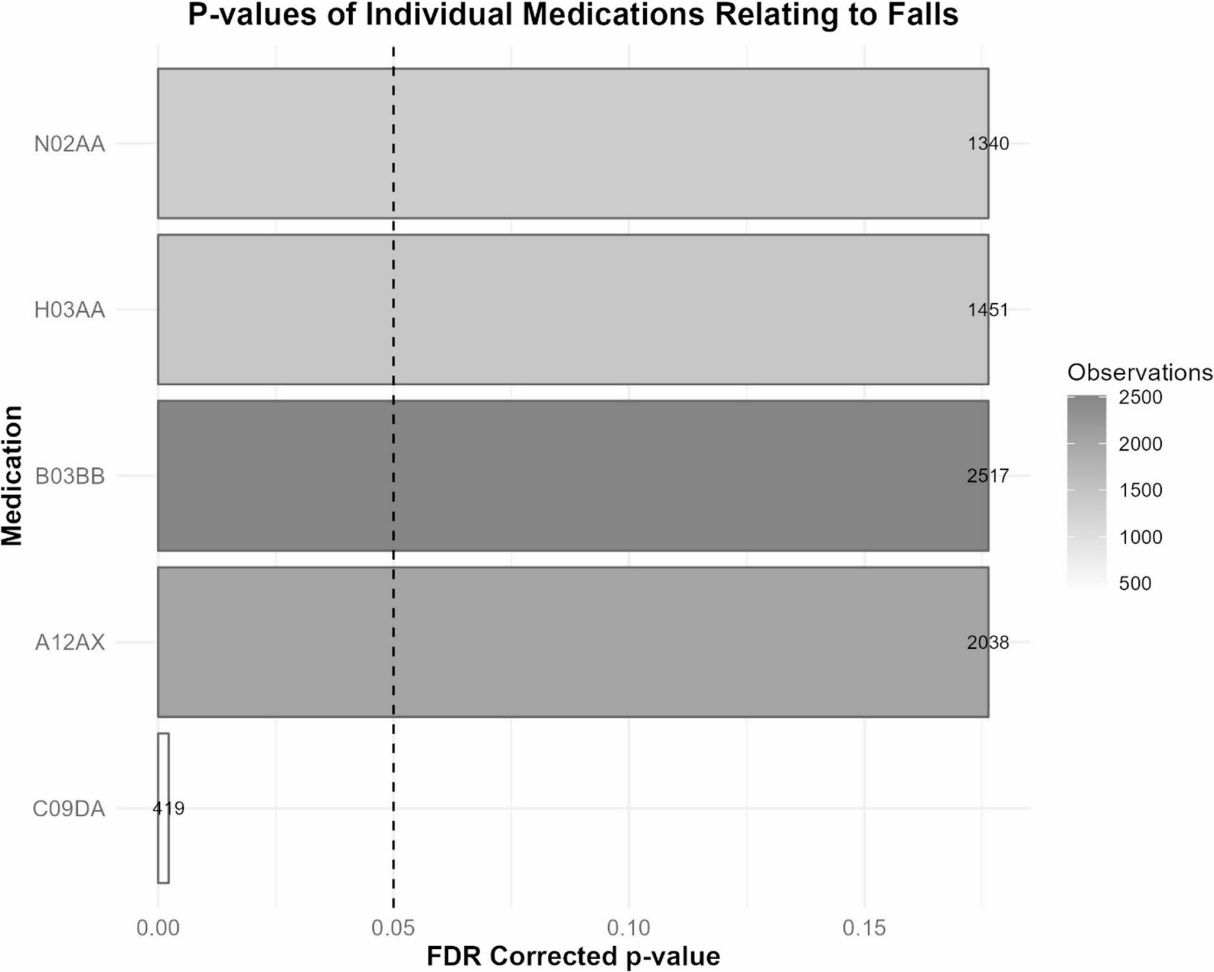
False discovery rate corrected p-values of the relationship between prescription medications and incidence of falls in the individual medication analysis. Vertical line represents FDR-corrected *p* = 0.05. The figure shows only those medications with uncorrected *p*-values < 0.05. Statistical outcomes for all medications can be found in supplemental Table 1. Also shown are the number of times (across time and participants) that each medication was prescribed, noted as “observations”, both in greyscale and numerically next to each bar

**Fig. 3:**
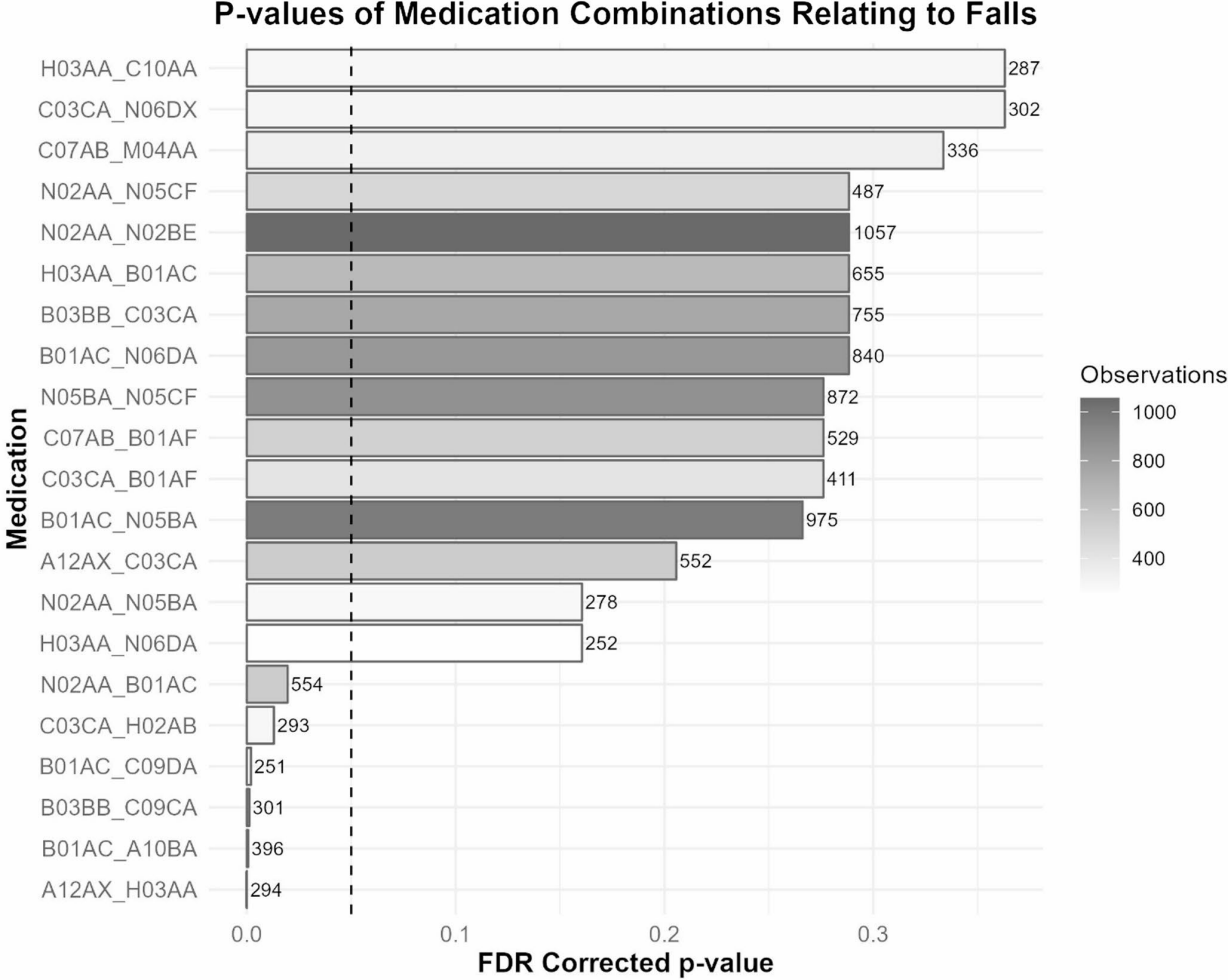
False discovery rate corrected *p*-values of the relationship between prescription medications and incidence of falls in the combination medication analysis. Vertical line represents FDR-corrected *p* = 0.05. The figure shows only those medications with uncorrected *p*-values < 0.05. Statistical outcomes for all medications can be found in supplemental Table 2. Also shown are the number of times (across time and participants) that each medication was prescribed, noted as “observations”, both in greyscale and numerically next to each bar

 Figures 2 and 3 show cGEE *p*-values and number of observations of medications for the individual and combination-medication analyses respectively (only medications with uncorrected *p*-values < 0.05 are shown). Full details regarding number of observations, number of participants, and output statistics (corrected and uncorrected *p*-values, OR, and 95% CI) for *all* individual and combination-medication models can be found in Supplemental Tables 1 and 2, respectively.

Of the 55 medications included in the individual-medication analysis, only C09DA (Angiotensin II receptor blockers (ARBs) and diuretics) was statistically significantly related to the observation of a consequential fall after accounting for age and total medication use (FDR corrected *p* = 0.022).

In the medication combination analysis, 163 medication combinations had at least 250 observations. Of these, 6 combinations were observed to have statistically significant relationships to fall incidence (Fig. 3, Table 2, and Supplemental Table 2). The most frequent medication class observed in this analysis was B01AC (platelet aggregation inhibitors excl. heparin), which was related to falls when prescribed with either N02AA (opioids), C09DA (ARBs and diuretics), or A10BA (blood glucose lowering drugs- Biguanides, Table 2). Other significant findings from the medication combination analysis included CO9DA & B03BB (folic acid), C03CA (High-Ceiling Diuretics- sulfonamides, plain) & H02AB (glucocorticoids), and finally A12AX (calcium, combinations with vitamin D and/or other drugs) & H03AA (thyroid hormones).

## Discussion

This report outlines relationships between injurious falls and prescription medications (both individually and in combination). Results showed that blood pressure reducing medications (e.g., C09DA) were related to consequential falls. We also observed that consequential falls were more frequently registered when platelet aggregation inhibitors (B01AC) were prescribed with blood pressure lowering drugs, biguanides, or opioids. These results provide novel information regarding possible direct and indirect relationships between medication use and injurious falls.

Drugs of the class C09DA (Angiotensin II receptor blockers (ARBs) and diuretics) were suggested to be related to the incidence of consequential falls. These medications are largely prescribed to reduce blood pressure and could increase the likelihood of experiencing a severe fall via acute orthostatic hypotension given the previously described relationships between hypotension and falls (Mol et al. 2019). Notably however, the specific relationship between hypertension medications and falls is complex. In particular, angiotension blockers have been shown to increase (Kamaruzzaman et al. 2010), have no effect (Canney et al. 2016), or even reduce (Di Stefano et al. 2015) orthostatic hypertension across varying samples of adults (for review, see: (Juraschek et al. 2024). As such, while it is plausible that ABRs could increase the risk of hypotension and thus falls, the specific causal mechanism between the observed relationship in the current study is speculative. Regardless of the mechanism, previous work has indicated that cardiovascular drugs are associated with increased fall risk (for meta-analysis, see (de Vries et al. 2018). More specifically, drugs of the class C09 (angiotensin II receptor blockers, combinations) and diuretics have been linked to falls in older adults, particularly in those 85 or above (see Fig. 1 in (Ming et al. 2022). The current analysis extends these findings in several ways. First, it links this medication specifically to falls with a reported consequence (e.g., an injury), rather than injuries that may or may not have been caused by a fall, which are a common outcome of interest in previous reports (Jung et al. 2023; Ming et al. 2022). Second, this link was identified from a broad list of medications, among which the only exclusion was number of times prescribed (to ensure robust model fits). Finally, the cGEE model currently used facilitates adjustment for time-stable confounders, such as genetics and sex. In other words, because individuals serve as their own “control” when periods with and without the medication are compared, possible time-stable confounding variables are accounted for.

Drug-interaction analyses showed that drugs in the class B01AC (platelet aggregation inhibitors excl. heparin), in combination with several other medications, were related to incidence of falls. B01AC medications include low dose aspirin (75 mg daily) and clopidogrel, both primarily prescribed as prophylactic antiplatelet agents to prevent ischemic cardiovascular disease. However, we propose that while this may be beneficial for many individuals, such medications could increase the chance that any falls that occur could be injurious in nature. For example, we observed that injurious falls were more common in people prescribed medications in the class B01AC and C09DA. As noted above, drugs in the class C09DA are often prescribed to reduce blood pressure, which may have the side effect of orthostatic hypotension. As such, the combination of medication what could increase risk of falls (C09DA), with one that could increase risk of serious hematoma after a fall (B01AC), may have underpinned our finding, and should be considered when prescribing medication. In line with this result, a previous study showed that individuals who were prescribed medications that can cause orthostatic hypotension (but not psychotropic fall-risk medications) were associated with fall-related hospital admissions in individuals with polypharmacy (> 9 medications) (Ryan-Atwood et al. 2017). Also, a recent double-blind study showed that increased rates of aspirin, a blood thinning agent, increased risk of serious falls compared to placebo (Barker et al. 2022). Together, these findings are consistent with a recent report questioning the use of low-dose aspirin as a primary preventative treatment for older adults (McNeil et al. 2018).

Similarly, fall incidence was more likely when B01AC medications were prescribed alongside drugs of the class A10BA (biguanides), which are prescribed to treat diabetes type 2. Diabetes increases fall risk in a number of ways, including peripheral neuropathy, hypoglycemia, and vision loss, among others (Freire et al. 2024). Therefore, prescription of platelet aggregation inhibitors for individuals with increased fall risk due to diabetes could increase the chance of falls being injurious in nature. This line of thought may also provide insight into other falls-medication interactions observed in this study. For example, we observed that prescription of diuretic medications (C03CA) in combination with glucocorticoids (H02AB) were linked to increase injurious fall-rate. Similarly to C09DA, diuretic medications could, in some circumstances, increase fall risk due to hypotension. Finally, glucocorticoids are commonly prescribed to treat conditions linked to fall risk, including chronic obstructive pulmonary disease (Roig et al. 2011). Therefore, while glucocorticoids are unlikely to increase fall-risk, prescription of diuretics (which could increase fall-risk) for people with already increased fall risk due to conditions associated with glucocorticoid use may result in an increase in risk for injurious falls. These findings should be considered with caution, and considering the limitations noted above. However, data indicate that prescription of blood thinning agents and blood pressure agents, in individuals with underlying conditions that can result in increased fall risk (as inferred by concomitant prescription of drugs such as biguanides and glucocorticoid), could result in an increased rate of injurious falls.

Finally, it was noted that individuals prescribed calcium/vitamin D preparations, along with thyroid hormones, more frequently experienced consequential falls. The rationale for this relationship is not fully clear. However, it is notable that these prescriptions, which are related to reduced bone density (A12AX), could indicate an underlying condition that is associated with increased risk of consequential falls. As with some examples above, such drug usage is unlikely to cause this increased injurious fall-risk, but could be used as a way to identify those at increased risk of such falls as a result of underlying conditions.

There was a notable lack of observed relationship between falls and commonly reported fall risk increasing drugs, such as antipsychotics, antidepressants, and benzodiazepines (Seppala et al. 2018; Seppala, Wermelink, Seppala et al. 2018a, b). This lack of finding could be for several reasons. First, our targeted approach only assessed participants with a confirmed fall (*n* = 440). While this approach led to a well-controlled and characterized sample, it reduced the number of potential medication-fall observations. For example, some psychotropic medications commonly linked to falls (e.g., antipsychotics [N05A]) & long-acting benzodiazepines [N05CD or N05BA]) were not prescribed enough in our cohort to allow for rigorous statistical analyses. This caveat aside, the lack of inclusion of medications here, due to low prescription rates in individuals who experienced falls, does provide interesting insight into the frequency of prescriptions in this Swedish cohort. Further, that the current analysis started with all eligible medications (rather than only the most commonly prescribed drugs, or the most common FRIDs) allows for a relatively unbiased assessment of possible drug-medication analyses.

Several limitations should be noted. First, as discussed above, confounding by indication is a possibility in the current study, such that the medication may be taken in response to a condition that increases fall risk, rather than the medication directly increasing fall risk. As such, care should be taken with interpretation of all results. Next, we linked falls to registered prescription of medications. As such, we cannot confirm that these medications were actually consumed at the timepoint provided. Third, within each 4-week window, it was not feasible to assess whether the medication or fall occurred first within each 4-week window. Fourth, while we believe the objective registration of falls by health professionals is a strength of the study, it is possible that other falls occurred but were not reported. Finally, while our sample contains a well-described cohort, larger cohorts may increase power to detect drug-falls relationships that were not observed in the current study.

## Conclusions

This manuscript utilizes rigorous and large Swedish medication and falls registries to relate the incidence of consequential falls to prescription of medications, both individually and in combination. In this cohort, we observed that cardiovascular medications (specifically that of the class C09DA), individually, and platelet aggregation inhibitor medications (B01AC), in combination with several other medications that may reflect underlying health conditions (e.g., opioids, blood pressure, biguanides), are related to an increased incidence of consequential falls. These results provide additional support for careful consideration of prescription of medications that could increase fall-risk (e.g., blood pressure medication, diuretics), in patients who are on platelet aggregation inhibitors (as this could increase the risk of a fall being injurious), or those who are already at increased risk for falls based on other underlying conditions (indicated by prescription of glucocorticoids or biguanides). However, current findings cannot provide information on the causal pathways between medications and falls. As such, additional work is necessary to (1) confirm the current findings, and (2) identify mechanisms of medication-related falls in older adults.

## Supplementary Information

Below is the link to the electronic supplementary material.

Supplementary Material 1

## Data Availability

Participant-level data are part of a National Swedish Registry and cannot be made publicly available.
