# Supplementary material for: Relating consequential falls to individual and combinations of prescription medication use in Swedish older adults
Source: Eur J Clin Pharmacol. 2026 Feb 6;82(3):71. doi: 10.1007/s00228-026-04000-2 (PMC12881012; doi:10.1007/s00228-026-04000-2)
Supplement: Supplementary file 1 — Supplementary Material 1 [file 228_2026_4000_MOESM1_ESM.docx]

**SUPPLEMENTAL CONTENT**

**Supplemental Figure 1:** Flow-diagram showing numbers of medications included in individual (left) and combination (right) medication analyses.

**
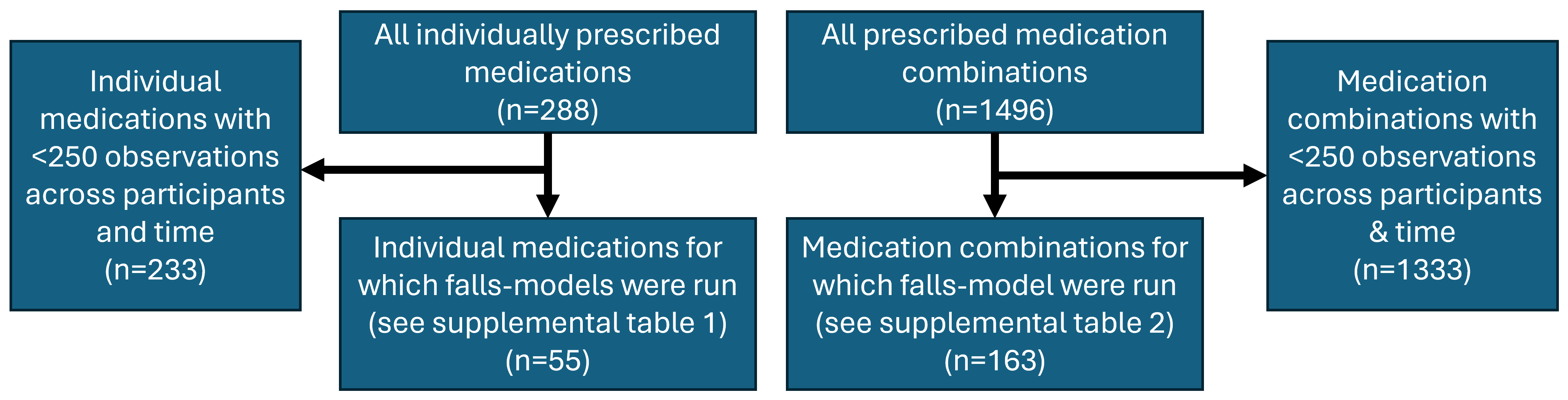
**

**Supplemental Figure 2**: Histogram of number of participants experiencing consequential falls.


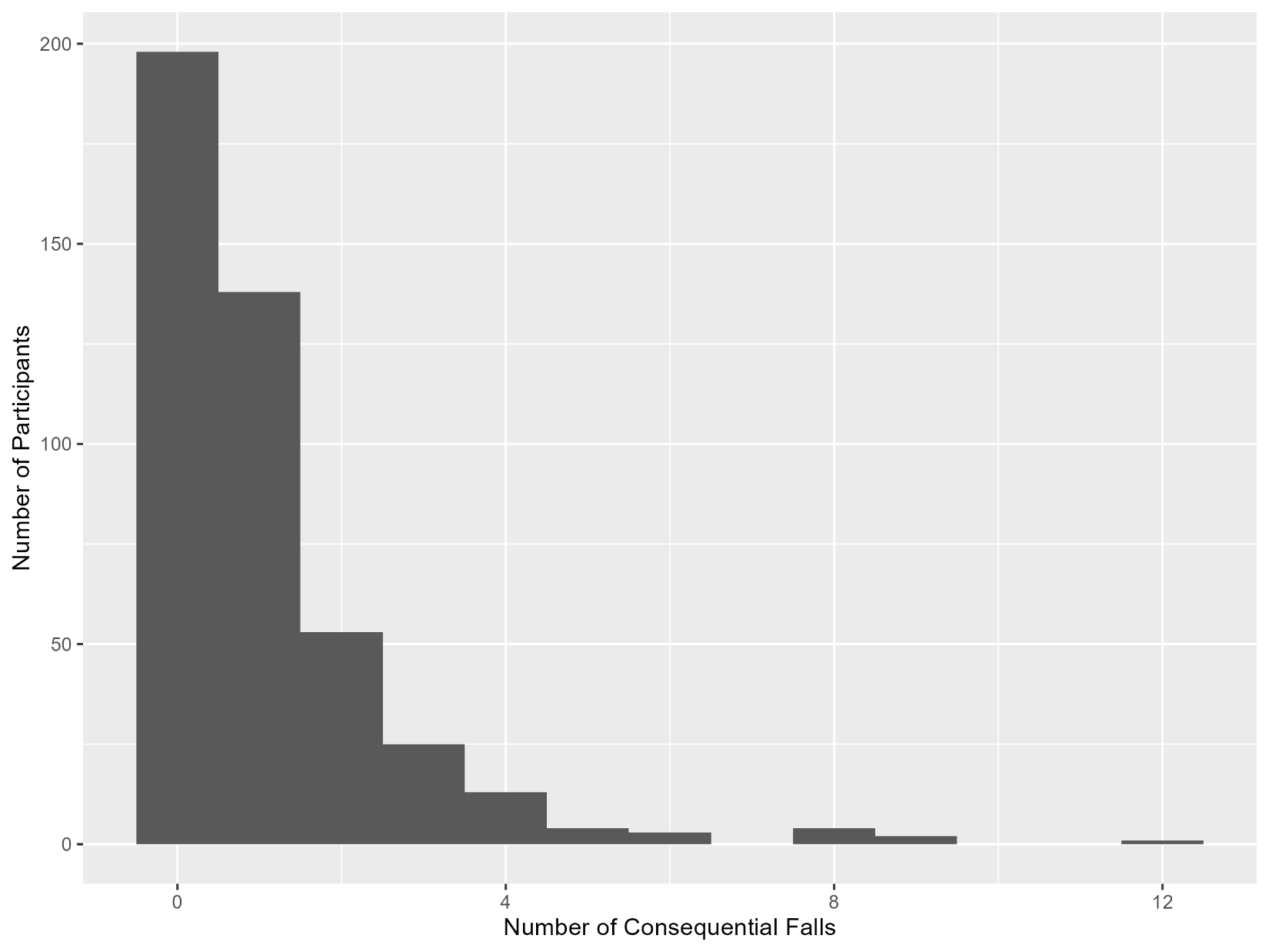


**Supplemental Table 1**: Details regarding all prescriptions included in the individual-medication analysis. 5-digit ATC code, the number of participants that were prescribed that medication, the total number of times the medication class was prescribed (across time and participants; minimum for inclusion = 250), uncorrected p-value of fall-medication class relationship, FDR-corrected p-value, model estimate, odds ratio, and 95% upper and lower confidence intervals of odds ratios are provided.

| Prescription Name | Participants Prescribed | Total Prescriptions | FDR Corrected p-value | Uncorrected p-value | Estimate | OR | OR Lower | OR Upper |
| --- | --- | --- | --- | --- | --- | --- | --- | --- |
| C09DA | 20 | 419 | 0.002 | 4.04E-05 | 2.146 | 8.55 | 3.07 | 23.83 |
| A12AX | 106 | 2038 | 0.176 | 0.014 | -0.584 | 0.56 | 0.35 | 0.89 |
| N02AA | 120 | 1340 | 0.176 | 0.007 | 0.778 | 2.18 | 1.24 | 3.81 |
| B03BB | 117 | 2517 | 0.176 | 0.011 | 0.795 | 2.21 | 1.20 | 4.10 |
| H03AA | 55 | 1451 | 0.176 | 0.016 | -1.826 | 0.16 | 0.04 | 0.71 |
| N02BE | 316 | 5379 | 0.554 | 0.071 | 0.437 | 1.55 | 0.96 | 2.49 |
| C03AA | 23 | 291 | 0.554 | 0.079 | 1.140 | 3.13 | 0.88 | 11.14 |
| S01ED | 26 | 256 | 0.554 | 0.081 | -0.673 | 0.51 | 0.24 | 1.09 |
| C07AB | 177 | 3987 | 0.645 | 0.113 | -0.494 | 0.61 | 0.33 | 1.12 |
| A06AB | 57 | 287 | 0.645 | 0.125 | 0.603 | 1.83 | 0.85 | 3.95 |
| D02AE | 87 | 476 | 0.645 | 0.145 | -0.486 | 0.62 | 0.32 | 1.18 |
| N06DA | 90 | 1859 | 0.645 | 0.152 | -0.556 | 0.57 | 0.27 | 1.23 |
| G04CA | 29 | 397 | 0.645 | 0.147 | 0.903 | 2.47 | 0.73 | 8.37 |
| N05BA | 161 | 2315 | 0.693 | 0.202 | 0.296 | 1.34 | 0.85 | 2.12 |
| Y92BA | 39 | 272 | 0.693 | 0.187 | -0.920 | 0.40 | 0.10 | 1.56 |
| B01AF | 45 | 727 | 0.693 | 0.190 | -0.531 | 0.59 | 0.27 | 1.30 |
| A02BC | 154 | 3250 | 0.755 | 0.252 | -0.329 | 0.72 | 0.41 | 1.26 |
| C10AA | 140 | 2862 | 0.755 | 0.261 | -0.383 | 0.68 | 0.35 | 1.33 |
| A10AD | 29 | 325 | 0.755 | 0.256 | -0.499 | 0.61 | 0.26 | 1.43 |
| A01AA | 51 | 306 | 0.770 | 0.280 | 0.400 | 1.49 | 0.72 | 3.08 |
| C08CA | 110 | 2079 | 0.779 | 0.340 | 0.309 | 1.36 | 0.72 | 2.57 |
| N02AB | 28 | 272 | 0.779 | 0.323 | 0.314 | 1.37 | 0.73 | 2.55 |
| N06AA | 12 | 285 | 0.779 | 0.314 | -0.595 | 0.55 | 0.17 | 1.76 |
| M05BA | 32 | 525 | 0.779 | 0.330 | -0.414 | 0.66 | 0.29 | 1.52 |
| B01AC | 193 | 4502 | 0.807 | 0.458 | -0.222 | 0.80 | 0.45 | 1.44 |
| H02AB | 67 | 946 | 0.807 | 0.408 | -0.406 | 0.67 | 0.25 | 1.74 |
| N05CF | 155 | 2713 | 0.807 | 0.460 | 0.186 | 1.20 | 0.74 | 1.97 |
| D02AX | 78 | 408 | 0.807 | 0.442 | -0.233 | 0.79 | 0.44 | 1.43 |
| C01DA | 66 | 1195 | 0.807 | 0.499 | -0.222 | 0.80 | 0.42 | 1.53 |
| M04AA | 31 | 650 | 0.807 | 0.384 | 0.348 | 1.42 | 0.65 | 3.10 |
| G03CA | 29 | 322 | 0.807 | 0.496 | 0.259 | 1.29 | 0.61 | 2.73 |
| C03DA | 22 | 323 | 0.807 | 0.487 | -0.423 | 0.66 | 0.20 | 2.16 |
| S01XA | 57 | 481 | 0.807 | 0.492 | -0.176 | 0.84 | 0.51 | 1.38 |
| C01AA | 23 | 482 | 0.807 | 0.396 | 0.824 | 2.28 | 0.34 | 15.31 |
| B01AA | 52 | 388 | 0.845 | 0.538 | -0.256 | 0.77 | 0.34 | 1.75 |
| C03CA | 156 | 2927 | 0.912 | 0.627 | -0.135 | 0.87 | 0.51 | 1.51 |
| C09CA | 67 | 1271 | 0.912 | 0.614 | -0.215 | 0.81 | 0.35 | 1.86 |
| N05AH | 20 | 342 | 0.912 | 0.653 | 0.182 | 1.20 | 0.54 | 2.66 |
| A06AD | 229 | 1354 | 0.912 | 0.663 | -0.088 | 0.92 | 0.62 | 1.36 |
| N05CM | 51 | 516 | 0.912 | 0.646 | 0.170 | 1.19 | 0.57 | 2.45 |
| Y92AD | 33 | 335 | 0.924 | 0.705 | -0.162 | 0.85 | 0.37 | 1.97 |
| N05AD | 22 | 283 | 0.924 | 0.692 | -0.247 | 0.78 | 0.23 | 2.65 |
| N06AX | 152 | 2892 | 0.973 | 0.898 | 0.035 | 1.04 | 0.61 | 1.77 |
| N05AX | 77 | 1211 | 0.973 | 0.893 | -0.069 | 0.93 | 0.34 | 2.54 |
| N03AX | 46 | 873 | 0.973 | 0.843 | -0.061 | 0.94 | 0.52 | 1.72 |
| N02AE | 55 | 390 | 0.973 | 0.816 | -0.083 | 0.92 | 0.46 | 1.85 |
| C09AA | 123 | 2424 | 0.973 | 0.866 | 0.058 | 1.06 | 0.54 | 2.08 |
| B03AA | 65 | 760 | 0.973 | 0.857 | -0.082 | 0.92 | 0.38 | 2.25 |
| N06DX | 73 | 1197 | 0.973 | 0.819 | 0.102 | 1.11 | 0.46 | 2.66 |
| A10BA | 45 | 910 | 0.973 | 0.789 | -0.131 | 0.88 | 0.34 | 2.29 |
| R03AC | 37 | 275 | 0.973 | 0.902 | -0.071 | 0.93 | 0.30 | 2.87 |
| B03BA | 162 | 3403 | 0.984 | 0.984 | 0.006 | 1.01 | 0.54 | 1.89 |
| N06AB | 167 | 3447 | 0.984 | 0.983 | 0.009 | 1.01 | 0.45 | 2.27 |
| A12BA | 31 | 414 | 0.984 | 0.959 | -0.025 | 0.98 | 0.37 | 2.54 |
| S01EE | 31 | 291 | 0.984 | 0.932 | -0.031 | 0.97 | 0.47 | 1.99 |

**Supplemental Table 2**: Details regarding all prescriptions included in the combination-medication analysis. 5-digit ATC code, the number of participants that were prescribed that medication, the total number of times the medication class was prescribed (across time and participants; minimum for inclusion = 250), uncorrected p-value of fall-medication class relationship, FDR-corrected p-value, model estimate, odds ratio, and 95% upper and lower confidence intervals of odds ratios are provided.

| Prescription Name | Participants Prescribed | Total Prescriptions | FDR Corrected p-value | Uncorrected p-value | Estimate | OR | OR Lower | OR Upper |
| --- | --- | --- | --- | --- | --- | --- | --- | --- |
| A12AX_H03AA | 16 | 294 | 1.53E-06 | 9.36E-09 | -3.823 | 0.02 | 0.01 | 0.08 |
| B01AC_A10BA | 22 | 396 | 0.001 | 8.53E-06 | 1.908 | 6.74 | 2.91 | 15.62 |
| B03BB_C09CA | 15 | 301 | 0.001 | 2.22E-05 | 1.436 | 4.20 | 2.17 | 8.17 |
| B01AC_C09DA | 11 | 251 | 0.002 | 4.94E-05 | 1.782 | 5.94 | 2.51 | 14.05 |
| C03CA_H02AB | 25 | 293 | 0.013 | 0.000 | -1.231 | 0.29 | 0.15 | 0.58 |
| N02AA_B01AC | 50 | 554 | 0.020 | 0.001 | 1.282 | 3.61 | 1.71 | 7.58 |
| N02AA_N05BA | 30 | 278 | 0.161 | 0.008 | 1.110 | 3.03 | 1.34 | 6.88 |
| H03AA_N06DA | 9 | 252 | 0.161 | 0.008 | -2.179 | 0.11 | 0.02 | 0.56 |
| A12AX_C03CA | 34 | 552 | 0.206 | 0.011 | -1.021 | 0.36 | 0.16 | 0.79 |
| B01AC_N05BA | 64 | 975 | 0.266 | 0.016 | 0.709 | 2.03 | 1.14 | 3.63 |
| C07AB_B01AF | 32 | 529 | 0.276 | 0.019 | -0.962 | 0.38 | 0.17 | 0.85 |
| C03CA_B01AF | 27 | 411 | 0.276 | 0.022 | -0.907 | 0.40 | 0.19 | 0.88 |
| N05BA_N05CF | 66 | 872 | 0.276 | 0.020 | 0.723 | 2.06 | 1.12 | 3.80 |
| N02AA_N02BE | 98 | 1057 | 0.288 | 0.026 | 0.673 | 1.96 | 1.08 | 3.54 |
| N02AA_N05CF | 46 | 487 | 0.288 | 0.029 | 0.578 | 1.78 | 1.06 | 2.99 |
| B03BB_C03CA | 39 | 755 | 0.288 | 0.031 | -0.845 | 0.43 | 0.20 | 0.92 |
| H03AA_B01AC | 24 | 655 | 0.288 | 0.030 | -1.236 | 0.29 | 0.10 | 0.89 |
| B01AC_N06DA | 39 | 840 | 0.288 | 0.032 | -1.145 | 0.32 | 0.11 | 0.91 |
| C07AB_M04AA | 15 | 336 | 0.334 | 0.039 | 1.033 | 2.81 | 1.05 | 7.49 |
| C03CA_N06DX | 20 | 302 | 0.363 | 0.047 | -1.284 | 0.28 | 0.08 | 0.98 |
| H03AA_C10AA | 11 | 287 | 0.363 | 0.046 | -0.761 | 0.47 | 0.22 | 0.99 |
| B03BB_B01AC | 53 | 1040 | 0.371 | 0.051 | 0.698 | 2.01 | 1.00 | 4.05 |
| C03CA_B01AC | 58 | 980 | 0.371 | 0.052 | 0.743 | 2.10 | 0.99 | 4.45 |
| C03CA_C10AA | 39 | 737 | 0.382 | 0.056 | -0.998 | 0.37 | 0.13 | 1.03 |
| C07AB_A06AD | 69 | 362 | 0.508 | 0.078 | -0.777 | 0.46 | 0.19 | 1.09 |
| C10AA_C08CA | 44 | 859 | 0.523 | 0.087 | 0.657 | 1.93 | 0.91 | 4.09 |
| B01AC_M04AA | 19 | 401 | 0.523 | 0.086 | 0.758 | 2.13 | 0.90 | 5.08 |
| N02AA_C08CA | 24 | 292 | 0.523 | 0.090 | 1.034 | 2.81 | 0.85 | 9.28 |
| C07AB_N02BE | 126 | 1820 | 0.546 | 0.097 | -0.491 | 0.61 | 0.34 | 1.09 |
| C07AB_C10AA | 71 | 1366 | 0.606 | 0.114 | -0.810 | 0.44 | 0.16 | 1.21 |
| C10AA_C01DA | 26 | 387 | 0.606 | 0.115 | -0.819 | 0.44 | 0.16 | 1.22 |
| C07AB_N05AX | 23 | 343 | 0.665 | 0.143 | 1.682 | 5.38 | 0.57 | 51.00 |
| B03BB_C08CA | 24 | 383 | 0.665 | 0.137 | -0.747 | 0.47 | 0.18 | 1.27 |
| N02BE_C01DA | 41 | 683 | 0.665 | 0.134 | -0.634 | 0.53 | 0.23 | 1.22 |
| C08CA_C09AA | 30 | 399 | 0.665 | 0.141 | 1.338 | 3.81 | 0.64 | 22.65 |
| N02BE_C08CA | 67 | 1060 | 0.677 | 0.150 | 0.503 | 1.65 | 0.83 | 3.28 |
| B03BB_A06AD | 53 | 291 | 0.825 | 0.192 | -0.610 | 0.54 | 0.22 | 1.36 |
| N02BE_B01AC | 134 | 1952 | 0.825 | 0.190 | 0.353 | 1.42 | 0.84 | 2.41 |
| N06AB_B03AA | 24 | 313 | 0.845 | 0.359 | 0.557 | 1.75 | 0.53 | 5.75 |
| A12AX_C07AB | 37 | 574 | 0.845 | 0.266 | -0.511 | 0.60 | 0.24 | 1.48 |
| A12AX_C10AA | 30 | 523 | 0.845 | 0.327 | -0.461 | 0.63 | 0.25 | 1.59 |
| A12AX_H02AB | 29 | 447 | 0.845 | 0.239 | -0.547 | 0.58 | 0.23 | 1.44 |
| A12AX_N05CF | 31 | 431 | 0.845 | 0.270 | -0.664 | 0.52 | 0.16 | 1.68 |
| A12AX_M05BA | 26 | 421 | 0.845 | 0.269 | -0.556 | 0.57 | 0.21 | 1.54 |
| C07AB_N02AA | 40 | 433 | 0.845 | 0.354 | 0.344 | 1.41 | 0.68 | 2.92 |
| C07AB_C03CA | 75 | 1346 | 0.845 | 0.280 | -0.392 | 0.68 | 0.33 | 1.38 |
| C07AB_N05CF | 70 | 843 | 0.845 | 0.281 | 0.390 | 1.48 | 0.73 | 3.00 |
| C07AB_C01DA | 42 | 712 | 0.845 | 0.361 | -0.430 | 0.65 | 0.26 | 1.64 |
| B03BB_N02BE | 89 | 1376 | 0.845 | 0.269 | -0.414 | 0.66 | 0.32 | 1.38 |
| B03BB_N05BA | 30 | 413 | 0.845 | 0.332 | 0.392 | 1.48 | 0.67 | 3.27 |
| B03BB_C09AA | 36 | 512 | 0.845 | 0.350 | 0.570 | 1.77 | 0.54 | 5.84 |
| N02BE_C10AA | 89 | 1278 | 0.845 | 0.206 | 0.528 | 1.70 | 0.75 | 3.84 |
| N02BE_N05BA | 102 | 1150 | 0.845 | 0.264 | 0.401 | 1.49 | 0.74 | 3.02 |
| N02BE_A10BA | 31 | 324 | 0.845 | 0.329 | 0.528 | 1.69 | 0.59 | 4.89 |
| N02BE_S01XA | 32 | 289 | 0.845 | 0.373 | -0.389 | 0.68 | 0.29 | 1.60 |
| C03CA_A06AD | 60 | 332 | 0.845 | 0.264 | -0.440 | 0.64 | 0.30 | 1.39 |
| C03CA_N06DA | 19 | 283 | 0.845 | 0.214 | 1.147 | 3.15 | 0.52 | 19.23 |
| C03CA_C03DA | 17 | 269 | 0.845 | 0.222 | -0.605 | 0.55 | 0.21 | 1.44 |
| H03AA_N05BA | 26 | 295 | 0.845 | 0.340 | -0.494 | 0.61 | 0.22 | 1.68 |
| H03AA_N05CF | 20 | 333 | 0.845 | 0.341 | 0.490 | 1.63 | 0.60 | 4.47 |
| C10AA_C09CA | 27 | 410 | 0.845 | 0.315 | -0.805 | 0.45 | 0.09 | 2.15 |
| C10AA_N06DA | 27 | 454 | 0.845 | 0.342 | -0.679 | 0.51 | 0.13 | 2.06 |
| C10AA_N05CF | 50 | 649 | 0.845 | 0.365 | -0.374 | 0.69 | 0.31 | 1.55 |
| C10AA_N06DX | 18 | 295 | 0.845 | 0.275 | 0.355 | 1.43 | 0.75 | 2.69 |
| C10AA_A10BA | 22 | 419 | 0.845 | 0.339 | -0.524 | 0.59 | 0.20 | 1.73 |
| B01AC_N05CF | 70 | 1039 | 0.845 | 0.217 | 0.512 | 1.67 | 0.74 | 3.76 |
| B01AC_C01DA | 37 | 730 | 0.845 | 0.314 | -0.495 | 0.61 | 0.23 | 1.60 |
| C09CA_N05CF | 23 | 254 | 0.845 | 0.228 | 0.661 | 1.94 | 0.66 | 5.67 |
| A06AD_N05CF | 57 | 305 | 0.845 | 0.369 | 0.294 | 1.34 | 0.71 | 2.55 |
| N06DA_N05CF | 25 | 294 | 0.845 | 0.303 | -0.533 | 0.59 | 0.21 | 1.62 |
| N05CF_N06DX | 20 | 319 | 0.845 | 0.284 | 0.653 | 1.92 | 0.58 | 6.35 |
| N05CF_C01DA | 24 | 345 | 0.845 | 0.345 | 0.444 | 1.56 | 0.62 | 3.91 |
| N02BE_N06DX | 48 | 565 | 0.847 | 0.390 | -0.418 | 0.66 | 0.25 | 1.71 |
| N02BE_M04AA | 19 | 291 | 0.847 | 0.386 | 0.713 | 2.04 | 0.41 | 10.22 |
| B01AC_H02AB | 27 | 266 | 0.847 | 0.387 | -0.346 | 0.71 | 0.32 | 1.55 |
| C07AB_H03AA | 17 | 407 | 0.848 | 0.395 | -0.788 | 0.45 | 0.07 | 2.80 |
| N06AB_N05CF | 55 | 870 | 0.856 | 0.404 | 0.341 | 1.41 | 0.63 | 3.14 |
| C07AB_H02AB | 23 | 303 | 0.860 | 0.412 | -0.509 | 0.60 | 0.18 | 2.03 |
| B01AC_N03AX | 17 | 318 | 0.866 | 0.420 | -0.400 | 0.67 | 0.25 | 1.77 |
| B03BB_C10AA | 30 | 597 | 0.869 | 0.427 | 0.519 | 1.68 | 0.47 | 6.04 |
| N06AB_N06DA | 35 | 556 | 0.872 | 0.433 | -0.420 | 0.66 | 0.23 | 1.88 |
| C07AB_N06DA | 23 | 495 | 0.875 | 0.440 | 1.406 | 4.08 | 0.12 | 144.67 |
| N02BE_M05BA | 23 | 345 | 0.900 | 0.458 | -0.462 | 0.63 | 0.19 | 2.14 |
| N06AB_C01DA | 19 | 438 | 0.900 | 0.503 | 0.297 | 1.35 | 0.56 | 3.21 |
| A12AX_C09CA | 16 | 254 | 0.900 | 0.603 | -0.261 | 0.77 | 0.29 | 2.06 |
| A12AX_N06DA | 25 | 450 | 0.900 | 0.487 | -0.341 | 0.71 | 0.27 | 1.86 |
| A12AX_N06DX | 17 | 286 | 0.900 | 0.638 | 0.332 | 1.39 | 0.35 | 5.56 |
| C07AB_B01AC | 81 | 1555 | 0.900 | 0.555 | 0.245 | 1.28 | 0.57 | 2.88 |
| C07AB_C08CA | 44 | 780 | 0.900 | 0.527 | 0.366 | 1.44 | 0.46 | 4.48 |
| C07AB_N05BA | 53 | 658 | 0.900 | 0.579 | 0.201 | 1.22 | 0.60 | 2.48 |
| C07AB_A10BA | 24 | 447 | 0.900 | 0.503 | 0.441 | 1.55 | 0.43 | 5.66 |
| C07AB_C01AA | 16 | 309 | 0.900 | 0.619 | 0.455 | 1.58 | 0.26 | 9.48 |
| N02AA_B03BB | 27 | 316 | 0.900 | 0.474 | 0.425 | 1.53 | 0.48 | 4.90 |
| N02AA_C03CA | 37 | 411 | 0.900 | 0.585 | 0.261 | 1.30 | 0.51 | 3.32 |
| N02AA_C10AA | 27 | 266 | 0.900 | 0.565 | 0.468 | 1.60 | 0.32 | 7.88 |
| B03BB_H03AA | 11 | 261 | 0.900 | 0.527 | 0.468 | 1.60 | 0.37 | 6.82 |
| B03BB_N06DA | 15 | 260 | 0.900 | 0.606 | 0.428 | 1.53 | 0.30 | 7.79 |
| B03BB_N06DX | 16 | 252 | 0.900 | 0.583 | -0.433 | 0.65 | 0.14 | 3.04 |
| B03BB_C01DA | 19 | 301 | 0.900 | 0.575 | -0.313 | 0.73 | 0.24 | 2.19 |
| N02BE_H03AA | 39 | 625 | 0.900 | 0.504 | -0.388 | 0.68 | 0.22 | 2.12 |
| N02BE_N03AX | 33 | 493 | 0.900 | 0.477 | -0.328 | 0.72 | 0.29 | 1.78 |
| N02BE_C09CA | 45 | 604 | 0.900 | 0.622 | -0.284 | 0.75 | 0.24 | 2.33 |
| N02BE_C09AA | 81 | 1237 | 0.900 | 0.636 | 0.175 | 1.19 | 0.58 | 2.47 |
| N02BE_N05CF | 98 | 1361 | 0.900 | 0.640 | -0.127 | 0.88 | 0.52 | 1.50 |
| C03CA_H03AA | 23 | 320 | 0.900 | 0.511 | 0.348 | 1.42 | 0.50 | 3.99 |
| C03CA_C09CA | 25 | 311 | 0.900 | 0.583 | 0.349 | 1.42 | 0.41 | 4.93 |
| C03CA_N05CF | 52 | 711 | 0.900 | 0.611 | 0.232 | 1.26 | 0.52 | 3.08 |
| C03CA_M04AA | 17 | 283 | 0.900 | 0.629 | 0.358 | 1.43 | 0.34 | 6.10 |
| C03CA_C01AA | 18 | 358 | 0.900 | 0.520 | 0.550 | 1.73 | 0.32 | 9.27 |
| N05AX_B01AC | 30 | 377 | 0.900 | 0.538 | -0.356 | 0.70 | 0.23 | 2.18 |
| N05AX_N05BA | 39 | 440 | 0.900 | 0.500 | 0.303 | 1.35 | 0.56 | 3.26 |
| N05AX_N05CF | 28 | 316 | 0.900 | 0.534 | 0.364 | 1.44 | 0.46 | 4.53 |
| B01AC_C09AA | 54 | 1133 | 0.900 | 0.545 | -0.338 | 0.71 | 0.24 | 2.13 |
| C08CA_N05CF | 39 | 674 | 0.900 | 0.581 | 0.358 | 1.43 | 0.40 | 5.10 |
| C09AA_N05CF | 41 | 653 | 0.900 | 0.631 | 0.158 | 1.17 | 0.62 | 2.23 |
| C09AA_A10BA | 18 | 263 | 0.900 | 0.567 | 0.487 | 1.63 | 0.31 | 8.62 |
| C10AA_B01AC | 84 | 1679 | 0.902 | 0.653 | -0.182 | 0.83 | 0.38 | 1.85 |
| C09AA_N06DA | 18 | 352 | 0.902 | 0.648 | -0.329 | 0.72 | 0.17 | 2.96 |
| B01AC_N06DX | 28 | 443 | 0.910 | 0.664 | -0.232 | 0.79 | 0.28 | 2.26 |
| N02BE_H02AB | 48 | 542 | 0.925 | 0.681 | 0.212 | 1.24 | 0.45 | 3.39 |
| N05BA_N06DA | 27 | 381 | 0.925 | 0.692 | -0.232 | 0.79 | 0.25 | 2.49 |
| N05BA_N06DX | 30 | 364 | 0.925 | 0.693 | 0.178 | 1.19 | 0.50 | 2.88 |
| A12AX_N02AA | 25 | 344 | 0.934 | 0.744 | -0.218 | 0.80 | 0.22 | 2.97 |
| A12AX_N05BA | 32 | 384 | 0.934 | 0.723 | 0.162 | 1.18 | 0.48 | 2.87 |
| N02BE_C03CA | 99 | 1547 | 0.934 | 0.734 | 0.131 | 1.14 | 0.54 | 2.43 |
| C03CA_C08CA | 27 | 429 | 0.934 | 0.731 | 0.199 | 1.22 | 0.39 | 3.78 |
| H03AA_C09AA | 15 | 309 | 0.934 | 0.723 | 0.133 | 1.14 | 0.55 | 2.39 |
| B01AC_C09CA | 32 | 540 | 0.934 | 0.741 | -0.162 | 0.85 | 0.33 | 2.22 |
| C08CA_N06DA | 21 | 404 | 0.934 | 0.707 | 0.252 | 1.29 | 0.35 | 4.79 |
| C09CA_N05BA | 23 | 294 | 0.934 | 0.745 | 0.214 | 1.24 | 0.34 | 4.50 |
| C09AA_C01DA | 26 | 404 | 0.935 | 0.751 | 0.224 | 1.25 | 0.31 | 5.00 |
| A12AX_B01AC | 46 | 823 | 0.941 | 0.792 | -0.122 | 0.88 | 0.36 | 2.19 |
| C07AB_B03BB | 46 | 873 | 0.941 | 0.823 | 0.115 | 1.12 | 0.41 | 3.08 |
| C07AB_C09CA | 40 | 724 | 0.941 | 0.790 | -0.168 | 0.85 | 0.25 | 2.91 |
| B03BB_N05AX | 19 | 283 | 0.941 | 0.818 | 0.238 | 1.27 | 0.17 | 9.68 |
| B03BB_N05CF | 42 | 740 | 0.941 | 0.826 | 0.067 | 1.07 | 0.59 | 1.94 |
| N02BE_A06AD | 126 | 707 | 0.941 | 0.778 | 0.074 | 1.08 | 0.64 | 1.80 |
| N02BE_B03AA | 41 | 415 | 0.941 | 0.813 | -0.122 | 0.89 | 0.32 | 2.43 |
| C03CA_C01DA | 30 | 438 | 0.941 | 0.806 | -0.083 | 0.92 | 0.47 | 1.79 |
| C10AA_C09AA | 44 | 818 | 0.941 | 0.794 | -0.136 | 0.87 | 0.31 | 2.42 |
| B01AC_A06AD | 83 | 470 | 0.941 | 0.781 | -0.094 | 0.91 | 0.47 | 1.76 |
| N05BA_A06AD | 57 | 301 | 0.941 | 0.820 | 0.078 | 1.08 | 0.55 | 2.13 |
| N05BA_C09AA | 35 | 575 | 0.941 | 0.775 | 0.168 | 1.18 | 0.37 | 3.75 |
| B01AC_C08CA | 52 | 939 | 0.962 | 0.850 | 0.085 | 1.09 | 0.45 | 2.61 |
| N06AB_H02AB | 19 | 271 | 0.966 | 0.884 | 0.072 | 1.07 | 0.41 | 2.85 |
| A12AX_B03BB | 27 | 485 | 0.966 | 0.888 | -0.094 | 0.91 | 0.25 | 3.36 |
| C07AB_C09AA | 55 | 971 | 0.966 | 0.889 | -0.054 | 0.95 | 0.44 | 2.03 |
| C07AB_N06DX | 19 | 308 | 0.966 | 0.879 | -0.150 | 0.86 | 0.13 | 5.91 |
| B03BB_B03AA | 24 | 261 | 0.966 | 0.880 | 0.129 | 1.14 | 0.21 | 6.08 |
| N02BE_B01AF | 28 | 345 | 0.966 | 0.885 | -0.063 | 0.94 | 0.40 | 2.21 |
| A12AX_N02BE | 77 | 1118 | 0.972 | 0.904 | -0.037 | 0.96 | 0.53 | 1.75 |
| A12AX_C09AA | 24 | 352 | 0.972 | 0.907 | 0.089 | 1.09 | 0.25 | 4.81 |
| A12AX_C08CA | 24 | 408 | 0.976 | 0.923 | 0.036 | 1.04 | 0.50 | 2.15 |
| C10AA_N05BA | 34 | 475 | 0.976 | 0.918 | -0.049 | 0.95 | 0.37 | 2.43 |
| B01AC_B03AA | 23 | 310 | 0.976 | 0.928 | 0.049 | 1.05 | 0.37 | 3.02 |
| N06AB_N06DX | 29 | 423 | 0.977 | 0.935 | 0.050 | 1.05 | 0.32 | 3.49 |
| N02BE_N06DA | 52 | 775 | 0.982 | 0.949 | 0.029 | 1.03 | 0.43 | 2.48 |
| N06DA_N06DX | 20 | 261 | 0.982 | 0.952 | 0.039 | 1.04 | 0.29 | 3.72 |
| N02BE_N05AX | 48 | 523 | 0.991 | 0.968 | 0.019 | 1.02 | 0.40 | 2.61 |
| C08CA_N05BA | 31 | 469 | 0.991 | 0.979 | -0.013 | 0.99 | 0.38 | 2.55 |
| C09AA_N06DX | 17 | 335 | 0.991 | 0.975 | -0.024 | 0.98 | 0.22 | 4.40 |
| C03CA_N05BA | 36 | 447 | 0.997 | 0.997 | 0.001 | 1.00 | 0.43 | 2.33 |
| C03CA_C09AA | 58 | 923 | 0.997 | 0.995 | 0.002 | 1.00 | 0.46 | 2.17 |
